# Heterodimeric BMP-2/7 Antagonizes the Inhibition of All-Trans Retinoic Acid and Promotes the Osteoblastogenesis

**DOI:** 10.1371/journal.pone.0078198

**Published:** 2013-10-30

**Authors:** Wenjuan Bi, Zhiyuan Gu, Yuanna Zheng, Xiao Zhang, Jing Guo, Gang Wu

**Affiliations:** 1 School/Hospital of Stomatology, Zhejiang University, Hangzhou, P.R. China; 2 School of Stomatology, Zhejiang Chinese Medical University, Hangzhou, P.R. China; 3 Department of Prosthodontics, Peking University School and Hospital of Stomatology, Beijing, P. R. China; 4 Department of Oral Cell Biology, Academic Centre for Dentistry Amsterdam (ACTA), Research Institute MOVE, VU University and University of Amsterdam, Amsterdam, The Netherlands; 5 Department of Oral Implantology and Prosthetic Dentistry, Academic Centre for Dentistry Amsterdam (ACTA), Research Institute MOVE, VU University and University of Amsterdam, Amsterdam, The Netherlands; INSERM U1059/LBTO, Université Jean Monnet, France

## Abstract

**Objectives:**

Hypervitaminosis A and alcoholism can result in a low mineral density and compromised regenerative capacity of bone, thus delaying implant osteointegration. The inhibitory effect of all-trans retinoic acid on osteoblastogenesis is considered to be one of the mechanisms. We hypothesized that heterodimeric bone morphogenetic protein-2/7 could antagonize all-trans retinoic acid and enhance osteoblastogenesis, with an aim to accelerate and enhance bone regeneration and implant osteointegration.

**Materials and Methods:**

We applied 5 ng/ml or 50 ng/ml bone morphogenetic protein-2/7 to restore the osteoblastogenesis of pre-osteoblasts (MC3T3-E1 cell line) that was inhibited by 1 µM all-trans retinoic acid. We evaluated the efficacy by assessing cell numbers (proliferation), alkaline phosphatase activity (a marker for early differentiation), osteocalcin (a marker for late differentiation), calcium deposition (a marker for final mineralization) and the expression of osteoblastogenic genes (such as Runx2, Collagen Ia, alkaline phosphatase and osteocalcin) at different time points.

**Results:**

All-trans retinoic acid significantly inhibited the expression of all the tested osteoblastogenic genes and proteins except alkaline phosphatase activity. In the presence of ATRA, 50 ng/ml bone morphogenetic protein-2/7 not only completely restored but also significantly enhanced all the osteoblastogenic genes and proteins. On the 28^th^ day, mineralization was completely inhibited by all-trans retinoic acid. In contrast, 50 ng/ml BMP-2/7 could antagonize ATRA and significantly enhance the mineralization about 2.5 folds in comparison with the control treatment (no ATRA, no BMP2/7).

**Conclusions:**

Heterodimeric bone morphogenetic protein-2/7 bears a promising application potential to significantly promote bone regeneration and implant osteointegration for the patients with hypervitaminosis A and alcoholism.

## Introduction

Sufficient bone volume and adequate bone quality are of paramount importance to achieve a rapid establishment of implants’ functions in dentistry and orthopedics. However, various adverse bone conditions such as low density and compromised self-healing capacity of bone can significantly compromise new bone regeneration and implant osteointegration, and thus delay the loading of implants. Such adverse bone conditions can be resulted from some harmful lifestyles, such as the alcoholism and hypervitaminosis A. Alcoholism exhibits very high prevalence: it affects more than 14 million people in the United States. A large body of evidence suggests that a correlation between low bone mass and chronic alcohol abuse [1] with increased fracture risk [2]. Animal studies showed that a alcohol consumption could decrease the new bone formation [3] and delay implant osteointegration [4] possibly by reducing the number and activity of osteoblasts [5]. The detrimental effect of alcohol was even more harmful in comparison with nicotine [6]. On the other hand, researchers have found that hypervitaminosis A caused accelerated bone resorption, bone fragility, and spontaneous fractures [7], [8]. The chronic and significant enhancement of all-trans retinoic acid (ATRA), a metabolite of vitamin A, can be one of the mechanisms for such phenomena. The significantly increased ATRA in serum may also account for the detrimental effects of alcoholism [9].

All-trans retinoic acid (ATRA) can induce bone resorption and osteoporosis [10]. The suppression of ATRA on bone metabolism can be mediated by both increasing the osteoclastic bone resorption [11] and decreasing the osteoblastic bone formation [12]. It has been established that ATRA can suppress the proliferation of pre-osteoblasts and inhibit the formation of a mineralized matrix. This effect may be due to the induction and maintenance of a partially differentiated non-proliferating state of osteoblasts by ATRA [13].

As robust osteoinductive agents, bone morphogenetic proteins (BMPs) are a group of growth factors under the superfamily of transforming growth factor-β (TGF-β). BMPs, particularly BMP-2 and BMP-7 can significantly enhance all the cellular events during osteoblastogenesis, such as migration, proliferation, differentiation and mineralization [14]. Consequently, BMPs have been extensively investigated to accelerate and enhance bone regeneration and implant osteointegration [15]. However, the effective doses of homodimeric BMPs in clinic to promote bone formation are extremely high (e.g. up to milligrams) [16], [17], which results in not only a substantial economic burden to patients but also a series of potential side effects, such as the overstimulation of osteoclastic activity and an ectopic bone formation in an unintended area [18], [19]. One alternative approach to solve the problem is to adopt more potent forms of BMPs. We have shown that heterodimeric BMP-2/7 induced osteoblastogenesis with significantly lower optimal concentrations but similar maximum effects [14]. Our in-vivo experiment also proved that low-dose heterodimeric BMP-2/7 facilitated more rapid bone regeneration in a better quality in peri-implant bone defects than the homodimeric BMPs [20].

In this study, we hypothesized that heterodimeric BMP-2/7 could antagonize the inhibitory effect of ATRA and enhance the osteoblastogenesis of pre-osteobalsts. We adopted a murine calvaria-derived cell line (MC3T3-E1), which is the precursor of functionalized osteoblast and is often used to examine the potency of osteoinductive agents like BMPs [21].

## Materials and Methods

### Study Design

In this study, we tested our hypothesis that heterodimeric BMP-2/7 could antagonize the inhibitory effect of ATRA and enhance the osteoblastogenesis of pre-osteobalsts. We treated the pre-osteoblasts with the following treatments: 1) no ATRA, no BMP-2/7; 2) 1 µM ATRA, no BMP-2/7; 3) 1 µM ATRA, 5 ng/ml BMP-2/7; 4) 1 µM ATRA, 50 ng/ml BMP-2/7; 5) no ATRA, 5 ng/ml BMP-2/7; 6) no ATRA, 50 ng/ml BMP-2/7. The concentrations of BMP2/7 and ATRA were determined basing on the previous studies. In our previous studies, 5 ng/ml was the low-threshold concentration and 50 ng/ml was the optimal concentration for BMP-2/7 to induce the ALP activity and OCN expression [14]. In addition, 1 µM ATRA has repeatedly been adopted to study the *in-vitro* effects of ATRA on osteogenic differentiation [11], [22], [23].

We evaluated their effects on the osteoblastogenesis by assessing cell numbers (as a proliferation index), alkaline phosphatase (ALP) activity (a marker for early differentiation), osteoclacin (OCN) (a marker for late differentiation), calcium deposition (a marker for final mineralization) and the expression of various osteoblastogenic genes.

### Cell Culture

MC3T3-E1 cells (ATCC; Chinese Academy of since, Shanghai, China) were cultured in a α-Minimum essential medium (α-MEM) containing 10% fetal bovine serum (FBS) (Gibco®, Invitrogen, Grand Island, NY, USA). The medium was changed every 3 days. Exponentially growing cells were plated at a final concentration of 1×10^4^ cells/well in 24-well plates for the cell proliferation assay, at a final concentration of 2×10^5^ cells/well in 6-well plates for the ALP activity assay, OCN detection and PCR analysis, or at a final concentration of 3×10^4^ cells/well in 48-well plates for alizarin red staining. After incubation for 24h, the cells were subjected to a low-serum medium (α-MEM containing 1% FBS) for another 24 h. Thereafter, the cells were treated with the different combinations of heterodimeric BMP-2/7 (R&D Systems, Inc., Minneapolis, MN, USA) and/or ATRA (Sigma-Aldrich, St. Louis, MO, USA). Triplicates per group were performed for each parameter per time point.

### Cell Viability and Proliferation Assay

To investigate the cell viability and proliferation of MC3T3-E1 cells, the cell numbers of each group was determined by the alamar Blue cell viability reagent (Invitrogen Corporation, Carlsbad, CA, USA) after the treatment for 1 day and 4 days. The fluorescent intensity was measured using a fluorescence spectrometer (SpectraMax M5 Molecular Devices, Sunnyvale, CA, USA) at EX 540 nm/EM 590 nm.

### ALP Activity Assay

To assess the early differentiation of pre-osteoblasts, the ALP activity and total protein content were measured after the treatment for 4 day and 7 days. The ALP activity in the cell lysate (Sigma-Aldrich, St. Louis, MO) was determined using LabAssay™ ALP colorimetric assay kit (Wako Pure Chemicals, Osaka, Japan). The total protein content was measured at 570 nm using a commercial BCA Protein Assay kit (Beyotime, China). The values were expressed as nmol p-NP/ug total protein/hour to present the ALP activity.

### OCN Expression Assay

To assess the terminal differentiation of pre-osteoblasts, the OCN secreted into the cell culture medium was determined. The cell supernatants were collected on the 4^th^ day and the 7^th^ day and centrifuged (10,000 rpm, 4°C, 5 min) before detection. The OCN concentrations in the supernatants were determined by ELISA using a mouse OCN EIA kit (Biomedical Technologies, Stoughton, MA, USA) [24].

### Alizarin Red Staining

We compared the mineralization possibility of MC3T3-E1 cells stimulated by BMP-2/7 and ATRA. For this purpose, cells were treated with mineralizing medium (10% FBS, 50 µM L-ascorbic acid, and 10 mM β-glycerophosphate; Sigma-Aldrich, St. Louis, MO, USA) [25] containing BMP-2/7 and ATRA. The medium was replaced every 3 days. On the 21^st^ and 28^th^ day, mineralized nodules were determined by the staining of alizarin red (Sigma-Aldrich, St. Louis, MO, USA) as previously described [25], [26]. Culture plates were photographed by NIS-Elements F2.20 (Nikon Eclipse 80i, Tokyo, Japan), and the calcified areas were then quantified using a software of Image-Pro Plus 6.0.

### Isolation of Total RNA and Real-time Fluorescence Quantitative Polymerase Chain Reaction (RT-qPCR) Analysis

On the 1^st^, the 4^th^ and the 7^th^ day, the total RNA was extracted from the cells using RNeasy Mini Kit and purified with RNase-Free DNase Set reagent (Qiagen, Germany) following the manufacturer’s instructions. Total RNA was reverse transcribed to cDNA using a kit of PrimeScript® RT Master Mix (Perfect Real Time, Takara, Japanese). RT-qPCR was performed using a PrimeScript® RT reagent Kit (Perfect Real Time, Takara, Japanese) according to the manufacturer’s instruction. Specific primers used for detecting mRNA transcripts of the Runx2, Collagen I, ALP, OCN, and β-actin gene are as shown in Table 1. Transcripts were normalized to the β-actin transcript levels. Calculate the n-fold upregulation for each gene of interest over the internal control gene (β-actin gene) according to the delt-delt-Ct method using the formula: 2^-[(CT gene of interest-CTinternal control)sample-(CT gene of interest-CT internal control)control]^ [27].

**Table 1:** Primer sequences for real-time quantitative polymerase chain reaction analysis of the expression of Runx2, collagen I, alkaline phosphatase (ALP) and osteocalcin (OCN) genes.

Gene	Accession No.	Primers (F = forward; R = reverse)
Akp2 (ALP)	NM_007431	F: 5′- TGCCTACTTGTGTGGCGTGAA -3′;
		R: 5′- TCACCCGAGTGGTAGTCACAATG -3′
Osteocalcin (OCN)	NM_007541	F: 5′- AGCAGCTTGGCCCAGACCTA -3′;
		R: 5′- TAGCGCCGGAGTCTGTTCACTAC -3′
Collagen I	NM_007742	F: 5′- ATGCCGCGACCTCAAGATG -3′;
		R: 5′- TGAGGCACAGACGGCTGAGTA -3′
Runx2	NM_009820	F: 5′- CACTGGCGGTGCAACAAGA -3′;
		R: 5′- TTTCATAACAGCGGAGGCATTTC -3′
β-actin	NM_007393	F: 5′- AGGAGCAATGATCTTGATCTT -3′;
		R: 5′- TGCCAACACAGTGCTGTCT -3′

### Statistical Analysis

Statistical comparisons among the results were made by one-way analysis of variance (ANOVA). Post Hoc comparisons were made using Bonferroni corrections. The level of significance was set at *p*<0.05. SPSS software (version 20) for a Windows computer system was employed for the statistical analysis.

## Results

**Figure 1:**
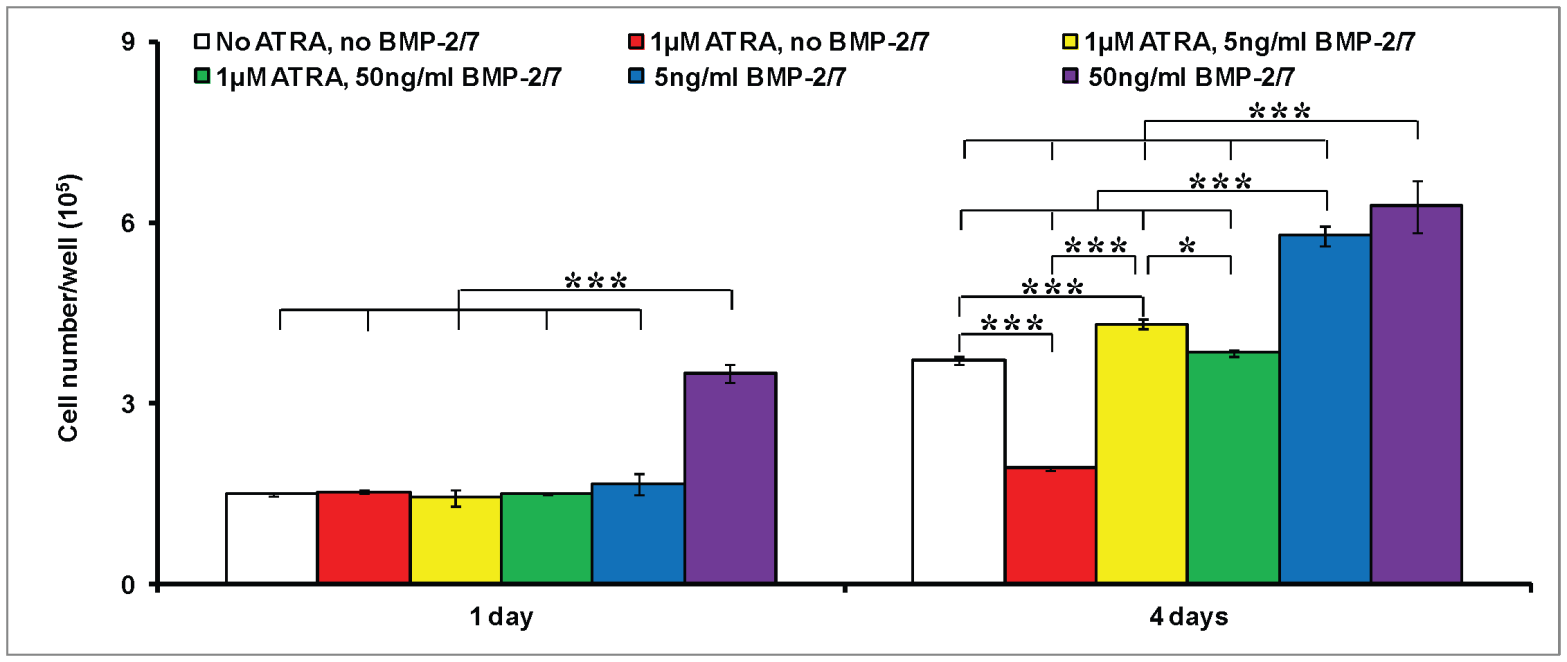
The cell numbers of murine calvarial pre-osteoblasts (MC3T3-E1 cells) per well under the different treatments. 1) no ATRA, no BMP-2/7; 2) 1 µM ATRA, no BMP-2/7; 3) 1 µM ATRA, 5 ng/ml BMP-2/7; 4) 1 µM ATRA, 50 ng/ml BMP-2/7; 5) no ATRA, 5 ng/ml BMP-2/7; 6) no ATRA, 50 ng/ml BMP-2/7 for 1 day and 4 days. All data are presented as mean values together with the standard deviation (SD). *: *p*<0.05, **: *p*<0.01, ***: *p*<0.001.

On the 1^st^ day, a significant increase in the cell numbers was detected only under the treatment of no ATRA, 50 ng/ml BMP-2/7 (Figure 1). Neither significant enhancement nor inhibition in cell proliferation was detected in the other groups. On the 4^th^ day, ATRA alone significantly decreased the cell numbers about 50%, which could be completely restored by 5 ng/ml or 50 ng/ml BMP-2/7. In contrast, 5 ng/ml or 50 ng/ml BMP-2/7 alone could result in a significantly higher cell number than the other treatments.

**Figure 2:**
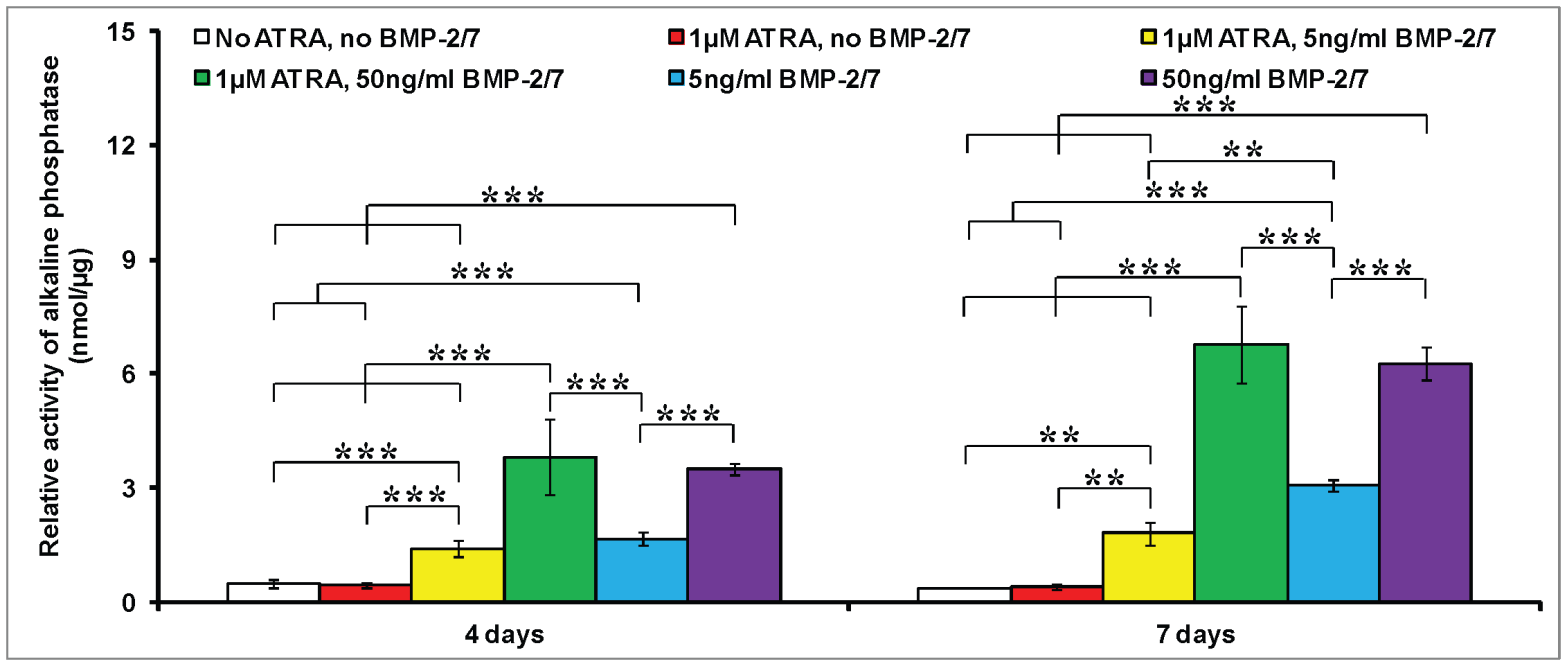
The activity of alkaline phosphatase (ALP) of murine calvarial pre-osteoblasts (MC3T3-E1 cells) under the different treatments. 1) no ATRA, no BMP-2/7; 2) 1 µM ATRA, no BMP-2/7; 3) 1 µM ATRA, 5 ng/ml BMP-2/7; 4) 1 µM ATRA, 50 ng/ml BMP-2/7; 5) no ATRA, 5 ng/ml BMP-2/7; 6) no ATRA, 50 ng/ml BMP-2/7 after for 4 day and 7 days. The ALP activity was normalized to total cellular protein content. All data are presented as mean values together with the standard deviation (SD). *: *p*<0.05, **: *p*<0.01, ***: *p*<0.001.

In contrast to the inhibitory effect of ATRA on cell proliferation, ATRA alone didn’t significantly influence the ALP activity on the 4^th^ day and the 7^th^ day (Figure 2). BMP-2/7 significantly increased the ALP activity in a dose-dependent manner irrespective of ATRA at both time points. On the 7^th^ day, 5 ng/ml and 50 ng/ml BMP-2/7 (with 1 µM ATRA) significantly increased the ALP activity 4.5 folds and 16.7 folds respectively in comparison with the treatment of no BMP-2/7, 1 µM ATRA.

**Figure 3:**
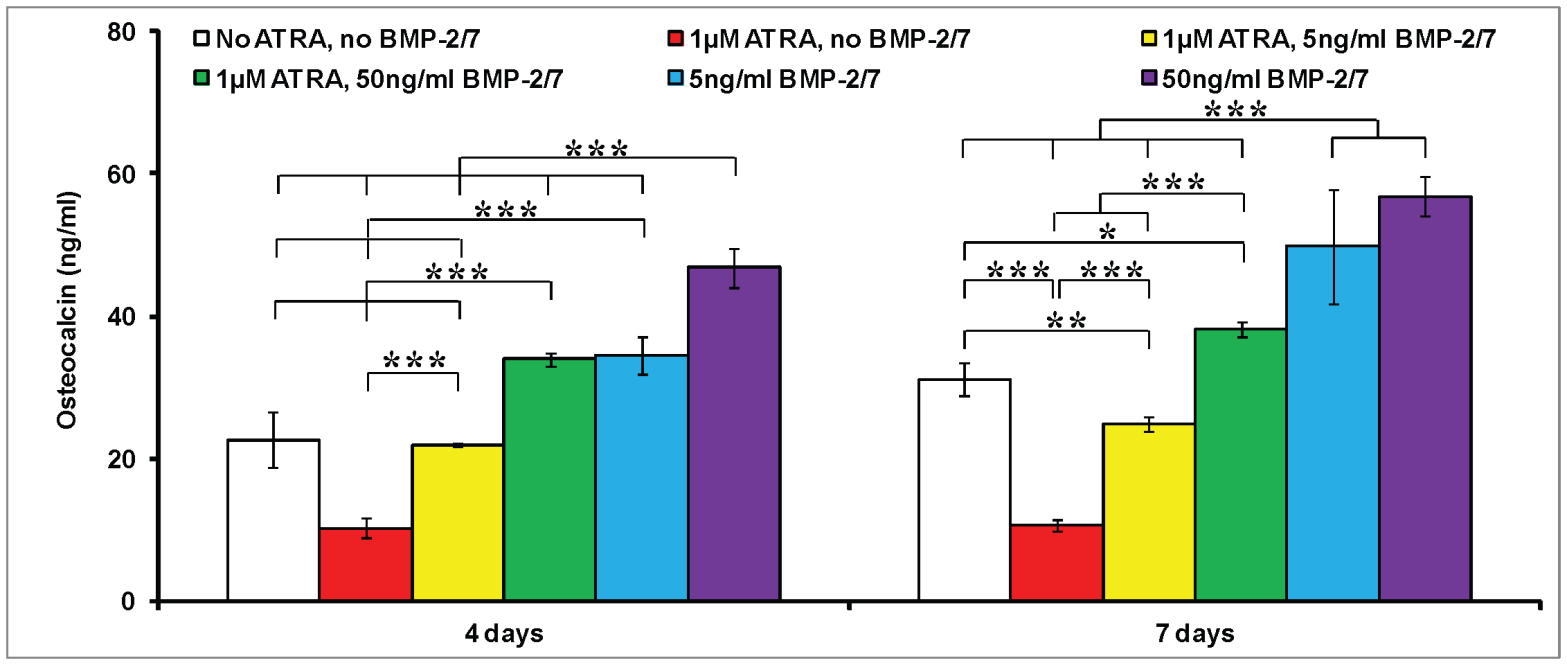
The expression of osteocalcin (OCN) of murine calvarial pre-osteoblasts (MC3T3-E1 cells) under the different treatments. 1) no ATRA, no BMP-2/7; 2) 1 µM ATRA, no BMP-2/7; 3) 1 µM ATRA, 5 ng/ml BMP-2/7; 4) 1 µM ATRA, 50 ng/ml BMP-2/7; 5) no ATRA, 5 ng/ml BMP-2/7; 6) no ATRA, 50 ng/ml BMP-2/7 for 4 days and 7 days. All data are presented as mean values together with the standard deviation (SD). *: *p*<0.05, **: *p*<0.01, ***: *p*<0.001.

In the absence of BMP-2/7 and ATRA, OCN expression in pre-osteoblasts significantly increased from the 4^th^ day to the 7^th^ day (Figure 3). Similar to its effects on cell proliferation, ATRA alone could significantly inhibit the OCN expression about 55% on the 4^th^ day and 66% on the 7^th^ day. On the 4^th^ day, 5 ng/ml BMP-2/7 antagonized the inhibitory effect of ATRA and completely restored the OCN expression. However, on the 7^th^ day, 5 ng/ml BMP-2/7 only restored about 70% OCN expression. In contrast, despite of the presence of ATRA, 50 ng/ml BMP-2/7 significantly enhanced the expression of OCN 1.5 times on the 4^th^ day and 1.23 times on the 7^th^ day respectively in comparison with the control treatment (no ATRA, no BMP-2/7). Albeit so, the BMP-2/7-induced OCN expression was significantly inhibited by ATRA at both time points.

**Figure 4:**
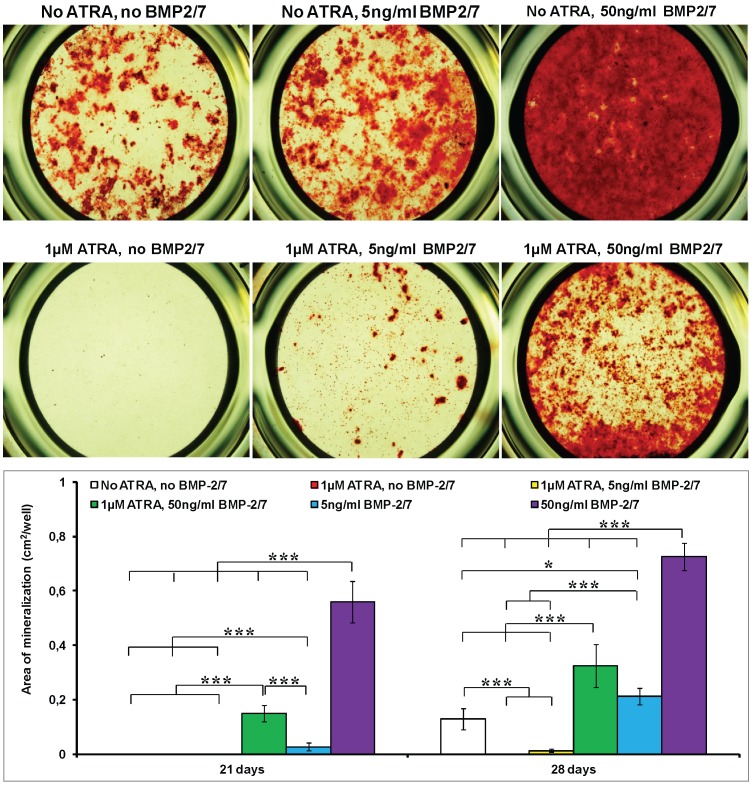
The mineralization of murine calvarial pre-osteoblasts (MC3T3-E1 cells) under the different treatments. 1) no ATRA, no BMP-2/7; 2) 1 µM ATRA, no BMP-2/7; 3) 1 µM ATRA, 5 ng/ml BMP-2/7; 4) 1 µM ATRA, 50 ng/ml BMP-2/7; 5) no ATRA, 5 ng/ml BMP-2/7; 6) no ATRA, 50 ng/ml BMP-2/7. (A) Light micrographs depicting the alizarin red staining on the 28^th^ day. (B) Graph depicting the calcification area on the 21^st^ day and the 28^th^ day. All data are presented as mean values together with the standard deviation (SD). *: *p*<0.05, **: *p*<0.01, ***: *p*<0.001.

On the 21^st^ day, the mineralization in cell matrix was found neither in the two groups without BMP-2/7 nor in the group of 1 µM ATRA, 5 ng/ml BMP-2/7 (Figure 4). 50 ng/ml BMP-2/7 alone resulted in the highest mineralization on the 21^st^ day. In the presence of ATRA, only 50 ng/ml BMP-2/7 was associated with significant mineralization. On the 28^th^ day, significant mineralization was also found in the control group (no ATRA, no BMP-2/7), which could be completely inhibited by ATRA (1 µM ATRA, no BMP-2/7). The mineralization area in the group of 1 µM ATRA, 5 ng/ml BMP-2/7 was still significantly lower than that in the control group (no ATRA, no BMP-2/7). In contrast, 50 ng/ml BMP-2/7 could antagonize ATRA and significantly enhance the mineralization about 2.5 folds in comparison with the control group (no ATRA, no BMP-2/7). 50 ng/ml BMP-2/7 alone resulted in the highest mineralization on the 28^th^ day.

**Figure 5:**
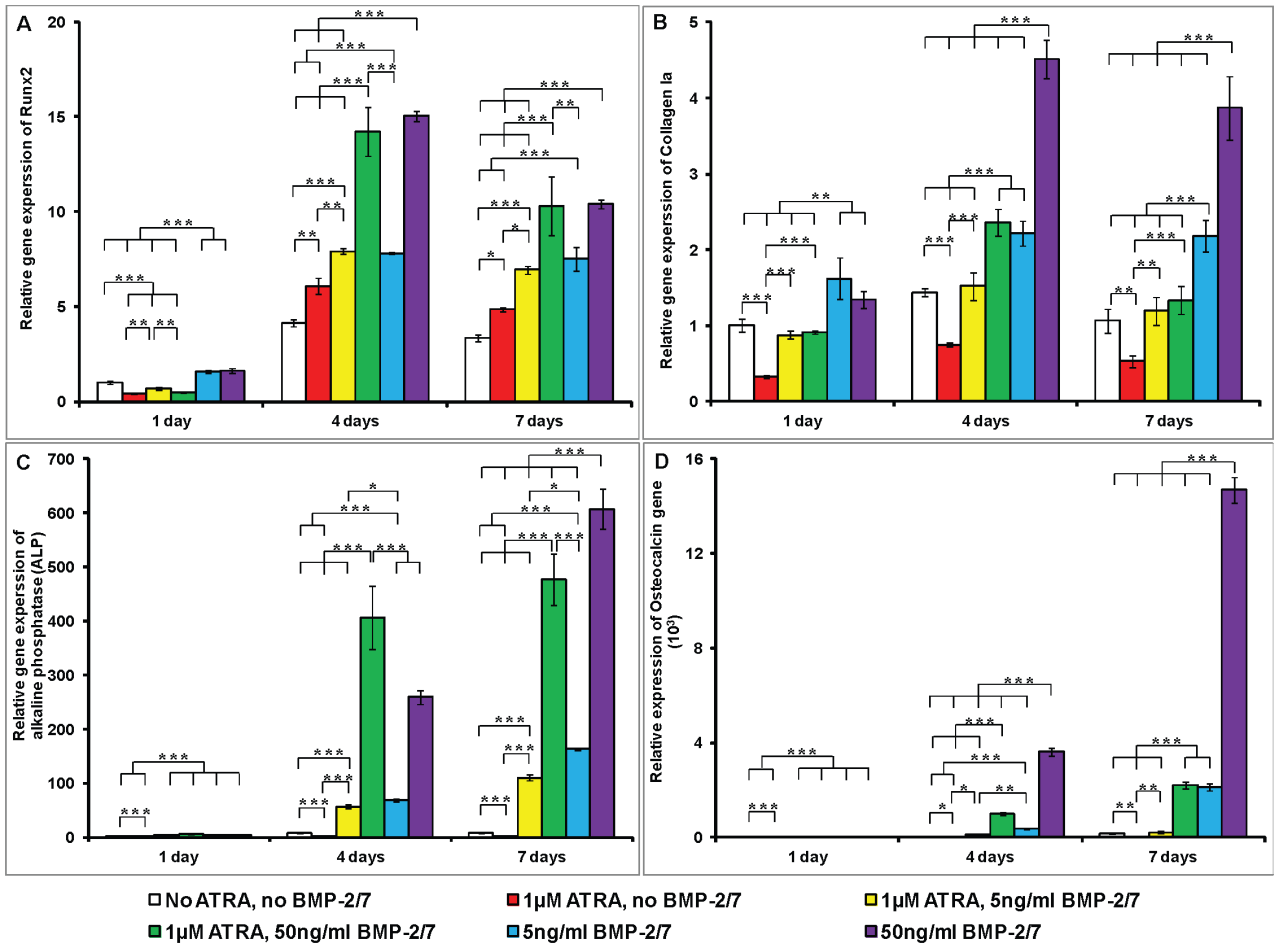
The relative expression of four osteogenic genes under the different treatments. 1) no ATRA, no BMP-2/7; 2) 1 µM ATRA, no BMP-2/7; 3) 1 µM ATRA, 5 ng/ml BMP-2/7; 4) 1 µM ATRA, 50 ng/ml BMP-2/7; 5) no ATRA, 5 ng/ml BMP-2/7; 6) no ATRA, 50 ng/ml BMP-2/7 for 1 day, 4 days and 7 days. (A) Runx2; (B) Collagen I; (C) Alkaline phosphatase; (D) Osteocalcin. The gene expression was first normalized to the corresponding β-actin gene expression for each sample. Then all the gene data were normalized to the gene data in control group on the 1^st^ day. All data are presented as mean values together with the standard deviation (SD). *: *p*<0.05, **: *p*<0.01, ***: *p*<0.001.

For all the treatment groups, the expression of Runx2 gene significantly increased from day 1 to day 4, while slightly decreased from day 4 to day 7. On the 1^st^ day, Runx2 expression was significantly suppressed by ATRA with or without BMP-2/7 (Figure 5A). The expression of Runx2 gene was, whereas, significantly enhanced by ATRA alone on the 4^th^ day and the 7^th^ day. BMP-2/7 significantly increased the expression of Runx2 gene in a dose-dependent manner. On the 4^th^ day and the 7^th^ day, ATRA didn’t significantly influence the expression of Runx2 that was induced by 5 or 50 ng/ml BMP-2/7. The expression of Collagen Ia gene was significantly suppressed by ATRA alone at the three time points (Figure 5B). 5 ng/ml BMP-2/7 could completely restore the expression of Collagen Ia gene that was inhibited by ATRA at all time points. 50 ng/ml BMP-2/7 could also restore the expression of Collagen Ia gene on the 1^st^ day and the 7^th^ day, and significantly enhance it on the 4^th^ day. ATRA could significantly decrease the gene expression of Collagen Ia that was induced by 5 or 50 ng/ml BMP-2/7 at all the time points. Different from the ALP activity, the expression of ALP gene was significantly suppressed by ATRA alone at all the three time points. In the presence of ATRA, 5 ng/ml and 50 ng/ml could significantly enhance ALP gene expression (Figure 5C) in comparison with the two groups without BMP-2/7. On the 4^th^ day, the highest expression of ALP gene was detected in the group of 1 µM ATRA, 50 ng/ml BMP-2/7. On the 7^th^ day, the highest expression of ALP gene was detected in the group of no ATRA, 50 ng/ml BMP-2/7. The expression of OCN gene was also significantly suppressed by ATRA alone at all the three time points. In the presence of ATRA, 5 ng/ml BMP-2/7 completely restored the expression of OCN gene, and 50 ng/ml could further significantly enhance the expression of OCN gene (Figure 5D). BMP-2/7 could significantly enhance the gene expression of OCN in a time-course and dose-dependent manner irrespective of ATRA. At both time points, ATRA significantly inhibited the expression of OCN gene that was induced by 5 or 50 ng/ml BMP-2/7.

## Discussion

Rapid restoration of bone defects and early loading of implants have been pursued in the field of orthopedics and dentistry. However, the goal becomes very difficult to achieve in the patients with low bone density and compromised self-healing capacity caused by the dietary-accumulated ATRA. In this study, we found that heterodimeric BMP-2/7, a potent osteoinductive cytokine, could antagonize the inhibitory effect of ATRA and enhance the osteoblastogenesis of pre-osteoblasts. Consequently, heterodimeric BMP-2/7 is very promising to accelerate and enhance bone regeneration and implant osteointegration for these patients.

ATRA, an active metabolite of vitamin A, is involved in bone formation [28]. ATRA regulates the gene expression via its receptors. There are two families of retinoic acid receptors, RARs and RXRs, each of which has three isotypes (α,β,γ). In consistency with the previous studies [29], [30], ATRA significantly inhibited cell proliferation (Figure 1), OCN expression (Figure 3) and mineralization (Figure 4), but did not influence the ALP activities (Figure 2). Coordinate gene expression patterns showed that ATRA treatment slowed the proliferation of MC3T3-E1 cells and inhibited the formation of a mineralized matrix [13]. This effect may be due to the induction and maintenance of a partially differentiated non-proliferating state. Until now, the molecular mechanisms for the effects of ATRA on the osteoblastogenesis of pre-osteoblasts remain largely unknown. Interestingly, Runx2, a key modulator for osteogenic differentiation, was significantly decreased only on the 1^st^ day, whereas significantly increased on the 4^th^ day and the 7^th^ day by ATRA (Figure 5A). Runx2 controls osteoblast proliferation and promotes a transition from a proliferative to a post-proliferative stage prior to osteoblast differentiation [31], [32]. It remains to be elucidated whether the significant up-regulation of Runx2 by ATRA could partially account for the significant down-regulation of cell proliferation. Besides, RA can induce cell growth arrest through the enhanced and prolonged MAPK signaling with BLR1 as a critical component in a positive feedback [33].

On the other hand, although the expression of Runx2 gene was significantly enhanced by ATRA alone, all the osteoblastic genes e.g. Col., ALP and OCN were significantly suppressed. In contrast, heterodimeric BMP2/7 alone could significantly enhance the ALP activity, OCN expression, mineralization and the all the selected osteogenic genes. ATRA could also suppress the BMP2/7-induced osteoblastogenesis of pre-osteoblasts. The mechanisms accounting for these phenomena remain ambiguous. It has been well established that homodimeric BMPs bind to transmembrane serine/threonine kinase receptors on the cell surface, triggering specific intracellular pathways that activate and influence gene transcription [34]. Activated BMP type I receptors phosphorylate Smad1, Smad5, and Smad8 (R-Smads), which then assemble into heteromeric complexes with Smad4 (Co-Smad) and translocate into the nucleus to regulate transcription of target genes, such as Runx2 [35]. In addition, the activated BMP receptors can also initiate Smad-independent signaling pathways, resulting in the activation of ERK, p38 and JNK [36], [37], [38]. Similarly, we also showed that heterodimeric BMP2/7 could significantly enhance the Id1 and Id2 (inhibitor of DNA binding 1 and 2, the canonical targets of BMP signaling) on the 4^th^ day (Figure S1). It was proposed that ATRA could down-regulate Id1 through promoting the degradation of phosphorylated Smad1 [23]. However, this mechanism seems not true in the pre-osteoblasts since ATRA could further promote the BMP2/7-induced Id1 and Id2. In consistency with Id1 and Id2, ATRA didn’t suppress the BMP2/7-induced Runx2. Consequently, ATRA might not promote the degradation of phosphorylated Smad1 in pre-osteoblasts. It might be plausible that ATRA interferes with the activation and functions of Runx2 possibly through inhibiting the phosphorylation and translocation to nuclei of Runx2. It is also important to note that the ALP activity remained unchanged under the treatment of ATRA alone. It has been shown that the induction of ALP activity was mediated through the activation of a Smad-independent signaling pathway p38 MAPK induced by BMP ligands [39]. Taken together, ATRA might inhibit the functions of Runx2 but enhance MAPK signaling, thereby facilitating the induction and maintenance of a partially differentiated non-proliferating state. The molecular mechanisms still need to be elucidated. The modulation of ATRA on the BMP signaling-related genes (such as Bambi, Mecom and Smurf1, figure S1) may provide some clues for further investigations.

In comparison with the homodimeric BMP-2 and BMP-7, heterodimeric BMP-2/7 was associated with significantly lower threshold and optimal concentrations, significantly earlier response [14]. Consequently, heterodimeric BMP-2/7 is very promising to efficiently accelerate and enhance bone regeneration and implant osteointegration. In this study, 5 and 50 ng/ml BMP-2/7 could antagonize the inhibitory effect of ATRA and restore the cell proliferation that was inhibited by ATRA (Figure 1). In the presence of ATRA, 5 ng/ml BMP-2/7 significantly enhanced the ALP activity and 50 ng/ml BMP-2/7 resulted in the highest ALP activity (Figure 2). Furthermore, 5 ng/ml BMP-2/7 can only partially rescue OCN that was inhibited by ATRA. In contrast, 50 ng/ml BMP-2/7 could further significantly enhance OCN 1.2 folds in comparison with the control treatment (no ATRA, no BMP-2/7). These results were compliant with our previous findings that BMP-2/7 induced the optimal ALP activity and OCN expression at the concentration of 50 ng/ml [14]. The findings from gene of ALP and OCN confirmed that BMP-2/7 could not only restore but also significantly enhance ALP and OCN that were inhibited by ATRA.

On the 21^st^ day, the mineralization in cell matrix was found neither in the two groups without BMP-2/7 nor in the group of 1 µM ATRA, 5 ng/ml BMP-2/7 (Figure 4). In the presence of ATRA, only 50 ng/ml BMP-2/7 was associated with detectable mineralization. These results indicated that 50 ng/ml heterodimeric BMP-2/7 could significantly accelerate mineralization even in the presence of ATRA. On the 28^th^ day, mineralization also occurred to the control group (no ATRA, no BMP-2/7), while such mineralization could be completely inhibited by ATRA. This result indicated that the up-regulation of Runx2 and maintenance of ALP by ATRA was insufficient for the final mineralization. 5 ng/ml BMP-2/7 only restored 8.5% mineralization, which suggested that the limited up-regulation of cell proliferation and ALP were neither sufficient to rescue ATRA-inhibited osteoblastogenesis. In contrast, 50 ng/ml BMP-2/7, that significantly enhanced all the osteoblastogenic genes and proteins (Figure 2, 3 and 5), not only restored but also significantly enhanced mineralization 2.5 folds (Figure 4). These results suggested that BMP-induced signaling could antagonize the inhibition of ATRA and significantly enhance osteoblastogenesis. Consequently, heterodimeric BMP-2/7 could not only significantly enhance but also accelerate the osteoblastogenesis, thereby bearing a promising application potential to facilitate bone regeneration and implant osteointegration for the patients with hypervitaminosis A and excessive alcohol consumption.

One of the limitations in this study was that we used a murine-derived pre-osteoblast cell line. It might be more instructive for clinic to investigate the effects of heterodimeric BMP-2/7 and ATRA on human primary cells. In addition, the effects of ATRA exhibited a large diversity on different cell types. Consequently, caution should be taken to extrapolate the results in other cell types.

## Supporting Information

Figure S1 **The microarray analysis of 27 BMP signaling-related genes in MC3T3-E1 cells that were treated with 1) no ATRA, no BMP2/7, 2) 1 µM ATRA, no BMP2/7, 3) no ATRA, 50 ng/ml BMP2/7 and 4) 1 µM ATRA, 50 ng/ml BMP2/7 for 4 days.** (A) Heat map and (B) table of fold changes (compared to the treatment of no ATRA, no BMP2/7).
(DOCX)
