# Supplementary material for: Heterodimeric BMP-2/7 Antagonizes the Inhibition of All-Trans Retinoic Acid and Promotes the Osteoblastogenesis
Source: PLoS One. 2013 Oct 30;8(10):e78198. doi: 10.1371/journal.pone.0078198 (PMC3813516; doi:10.1371/journal.pone.0078198)
Supplement: Figure S1 — The microarray analysis of 27 BMP signaling-related genes in MC3T3-E1 cells that were treated with 1) no ATRA, no BMP2/7, 2) 1 µM ATRA, no BMP2/7, 3) no ATRA, 50 ng/ml BMP2/7 and 4) 1 µM ATRA, 50 ng/ml BMP2/7 for 4 days. (A) Heat map and (B) table of fold changes (compared to the treatment of no ATRA, no BMP2/7). (DOCX) [file pone.0078198.s001.docx]

**Figure S1**

**B**

| RefSeq | Symbol | 1µM ATRA, no BMP2/7 | no ATRA, 50ng/ml BMP2/7 | 1µM ATRA, 50ng/ml BMP2/7 |
| --- | --- | --- | --- | --- |
| NM_007394 | Acvr1 | 1,12 | 1,10 | 1,38 |
| NM_007396 | Acvr2a | 1,28 | 0,88 | 1,38 |
| NM_009612 | Acvrl1 | 0,97 | 0,90 | 0,75 |
| NM_026505 | Bambi | 0,58 | 4,63 | 2,66 |
| NM_009758 | Bmpr1a | 1,28 | 1,32 | 1,22 |
| NM_007560 | Bmpr1b | 1,42 | 0,97 | 1,16 |
| NM_007561 | Bmpr2 | 2,35 | 2,17 | 2,38 |
| NM_009893 | Chrd | 0,48 | 0,85 | 0,51 |
| NM_007833 | Dcn | 4,38 | 5,43 | 3,01 |
| NM_010234 | Fos | 1,32 | 1,65 | 3,03 |
| NM_008046 | Fst | 1,39 | 2,66 | 3,14 |
| NM_008655 | Gadd45b | 1,87 | 1,93 | 3,18 |
| NM_010495 | Id1 | 1,38 | 5,31 | 10,06 |
| NM_010496 | Id2 | 3,27 | 10,13 | 14,22 |
| NM_010591 | Jun | 2,10 | 0,63 | 1,06 |
| NM_008416 | Junb | 0,26 | 1,56 | 1,51 |
| NM_007963 | Mecom | 6,82 | 0,88 | 4,89 |
| NM_008711 | Nog | 0,43 | 0,74 | 0,42 |
| NM_009821 | Runx1 | 6,02 | 1,47 | 2,81 |
| NM_008539 | Smad1 | 1,40 | 1,52 | 1,73 |
| NM_010754 | Smad2 | 1,37 | 1,46 | 1,75 |
| NM_016769 | Smad3 | 2,55 | 1,12 | 1,43 |
| NM_008540 | Smad4 | 1,51 | 1,26 | 1,27 |
| NM_008541 | Smad5 | 1,21 | 1,08 | 1,06 |
| NM_001042660 | Smad7 | 1,77 | 2,60 | 4,06 |
| NM_029438 | Smurf1 | 2,20 | 1,35 | 2,04 |
| M_009283 | Stat1 | 1,71 | 1,46 | 1,74 |

**Figure S1**

The microarray analysis of 27 BMP signaling-related genes in MC3T3-E1 cells that were treated with 1) no ATRA, no BMP2/7, 2) 1µM ATRA, no BMP2/7, 3) no ATRA, 50ng/ml BMP2/7 and 4) 1µM ATRA, 50ng/ml BMP2/7 for 4 days. (A) Heat map and (B) table of fold changes (compared to the treatment of no ATRA, no BMP2/7).

**Materials and methods for Figure S1**

Total RNA isolation, reverse transcription and RT^2^ profiler PCR array analysis

The total RNA was extracted from cells using RNeasy Mini kit and purified with the RNase-Free DNase Set (Qiagen, Germany) on the 4^th^ day, according to the manufacturer’s instructions. The cDNA samples were prepared from the isolated RNA using the RT^2^ first strand kit (Qiagen, Germany) according to the manufacturer’s protocols. RT^2^ profiler PCR array analysis was performed using the Mouse TGFβ/BMP signaling pathway PCR array (Cat. No. 330231, Qiagen, Germany), which combines the real-time PCR sensitivity and the multi-gene profiling capability of a microarray. This analysis was performed using a LightCycler^®^ 480 (Roche Diagnostics). Relative gene expression values were analyzed using the Superarray web-based software performing all delt-delt-Ct based fold-change calculations.
